# Effects of dietary supplementation with a standardized aqueous extract of *Terminalia chebula* fruit (AyuFlex^®^) on joint mobility, comfort, and functional capacity in healthy overweight subjects: a randomized placebo-controlled clinical trial

**DOI:** 10.1186/s12906-017-1977-8

**Published:** 2017-10-02

**Authors:** H. L. Lopez, S. M. Habowski, J. E. Sandrock, B. Raub, A. Kedia, E. J. Bruno, T. N. Ziegenfuss

**Affiliations:** 1 Division of Sports Nutrition and Exercise Science, The Center for Applied Health Sciences, 4302 Allen Road, Suite 120, Stow, OH 44224 USA; 2 Huntington College of Health Sciences, 117 Legacy View Way, Knoxville, TN 37918 USA

**Keywords:** *Terminalia chebula*, Joint health, AyuFlex, Connective tissue, Knee, Exercise capacity, Joint mobility, Chebulagic acids, Chebulinic acid, Anti-inflammatory, Cartilage oligomeric protein, Western Ontario McMaster universities arthritis index

## Abstract

**Background:**

Joint and connective tissue integrity, comfort and function are paramount to optimal performance in exercise, recreational and occupational activities. The fruit of *Terminalia chebula* has been used extensively in various traditional health systems for different ailments, with additional preclinical and clinical data demonstrating antioxidant and anti-inflammatory potential. The aim of this study was to evaluate the effects of a standardized aqueous extract of *Terminalia chebula* fruit (AyuFlex®) dietary supplementation on joint mobility, comfort, and functional capacity in healthy overweight subjects.

**Methods:**

One-hundred and five (105) overweight, apparently healthy male and female subjects (35–70 years of age) were pre-screened and randomized to one of three groups for 84 days: placebo, AyuFlex1 (250 mg twice daily) or AyuFlex2 (500 mg twice daily) in a randomized, double-blind, placebo-controlled design. A two-week placebo lead-in period was used to improve data quality/validity. All subjects had no knee joint discomfort at rest, but experienced knee joint discomfort only with activity/exercise of at least 30 on 100 mm Visual Analog Scale (VAS). Primary outcome measures included symptoms of joint health and function as measured by modified-Knee Injury & Osteoarthritis Outcomes Score (mKOOS) global & modified-Western Ontario and McMaster Universities Arthritis Index (mWOMAC) subscales (discomfort, stiffness and function). Secondary outcomes included VAS questionnaires on overall/whole-body joint health, low back health, knee mobility, willingness and ability to exercise, 6-min walk test for distance and range of motion (ROM) of pain-free knee flexion/extension. Tertiary outcome measures included inflammatory (high sensitivity C-reactive protein (hsCRP), tumor necrosis factor (TNF)-α) and extracellular matrix (ECM)/Connective Tissue (COMP) biomarkers, and safety (vital signs and blood markers) & tolerability (Adverse Event (AE)/ side effect profiles).

**Results:**

Compared to placebo, at day 84 AyuFlex® treatment significantly: 1) improved mKOOS global scores in AyuFlex1 + AyuFlex2 (*P* = 0.023), and improved total and physical function subscale of mWOMAC relative to baseline, 2) improved VAS scores for Knee Discomfort with activity/exercise in AyuFlex1 + AyuFlex2 (*P* = 0.001) relative to baseline, 3) improved VAS scores for whole-body joint function in AyuFlex1 + AyuFlex2 (*P* < 0.029) relative to baseline, 4) improved VAS score for decreased knee joint soreness following leg extension challenge for AyuFlex1 (*P* = 0.022) and AyuFlex2 (*P* = 0.043) relative to baseline, 5) improved 6-min walk performance distance covered (*P* = 0.047) and VAS discomfort (*P* = 0.026) post-6 min walk in AyuFlex1 + AyuFlex2 relative to baseline, 6) and tended to decrease COMP levels in AyuFlex1 + AyuFLex2 (*P* = 0.104) relative to baseline. All biomarkers of safety remained within normative limits during the study. Low back health tended to improve in the AyuFlex1 and AyuFlex2 group, but failed to reach significance relative to placebo group.

**Conclusions:**

AyuFlex® improved mKOOS global scores, knee joint discomfort with activity/exercise, 6-min walk test distance covered and discomfort post-6 min walk test, overall whole-body joint function, knee soreness following leg extension resistance exercise in a healthy, overweight population, without AE. Differences between 250 mg/BID and 500 mg/BID were non-significant for most of the outcome measures, validating the efficacy of the lower dose.

**Trial registration:**

ClinicalTrials.gov identifier NCT02589249; October 26, 2015.

## Background

Joint discomfort may result from various medical disorders, or simply as a response to exercise and physical activity in some populations. Osteoarthritis (OA) is a degenerative joint disease, characterized by focal and progressive loss of the hyaline cartilage of joints with joint space narrowing, and underlying bony changes (osteophytes and bony sclerosis) [1]. As the most common form of arthritis in the United States, OA affects 13.9% of adults aged 25 years and older and 33.6% (12.4 million) of those 65 and older [2]. Current nonsurgical treatments for OA are aimed at reducing pain, and include the use of oral non-steroidal anti-inflammatory drugs (NSAIDs) and injectable corticosteroids [1].

A wide variety of populations are subject to exercise-related joint pain, especially knee pain. For example, this is true of competitive swimmers [3], workers whose jobs involve significant outdoor activity [4], and overweight and obese individuals [5]. NSAIDs and topical medications are commonly used to treat exercise-related pain, although some data suggest that use of NSAIDs may actually be detrimental when exercise-induced muscle damage is present due to impairment of satellite cell activity [6].

While NSAIDs and corticosteroids have displayed efficacy in the treatment of OA, low back pain (LBP) and exercise-related pain, long-term use is associated with potentially serious adverse effects. These include dyspepsia, ulcers, bleeding, and 2–6 times increased risk of gastrointestinal complications with NASIDs. [7–9] For injectable corticosteroids, serious adverse effects include joint infection, nerve damage, thinning of skin and soft tissue around the injection site, tendon weakening or rupture, as well as osteoporosis or osteonecrosis of nearby bone [10].

The fruit of *Terminalia chebula* Retz. (Fam. Combretaceae) has been extensively used in Ayurvedic, Unani and Iranian medicine as a remedy against various human ailments [11, 12]. Human clinical research has demonstrated that *T. chebula* fruit has antioxidant and anti-inflammatory activity [13] which is relevant since oxidative stress [14, 15] and inflammation [16, 17] have contributory roles in OA, LBP and exercise-related joint discomfort.

The tannins are most prevalent at 32%–56%, and include gallic acid, ellagic acid, chebulic acid, chebulinic acid, punicalagin, and tannic acid. The flavonoids include quercetin, catechin, and kaempferol. Saccharides are present at 6%–9%, and include D-glucose, D-fructose, and saccharose. Quinic acid at 1.5%, and shikimic acid at 2% are the prevalent fruit acids [1–3, 19].

**Fig. 1:**
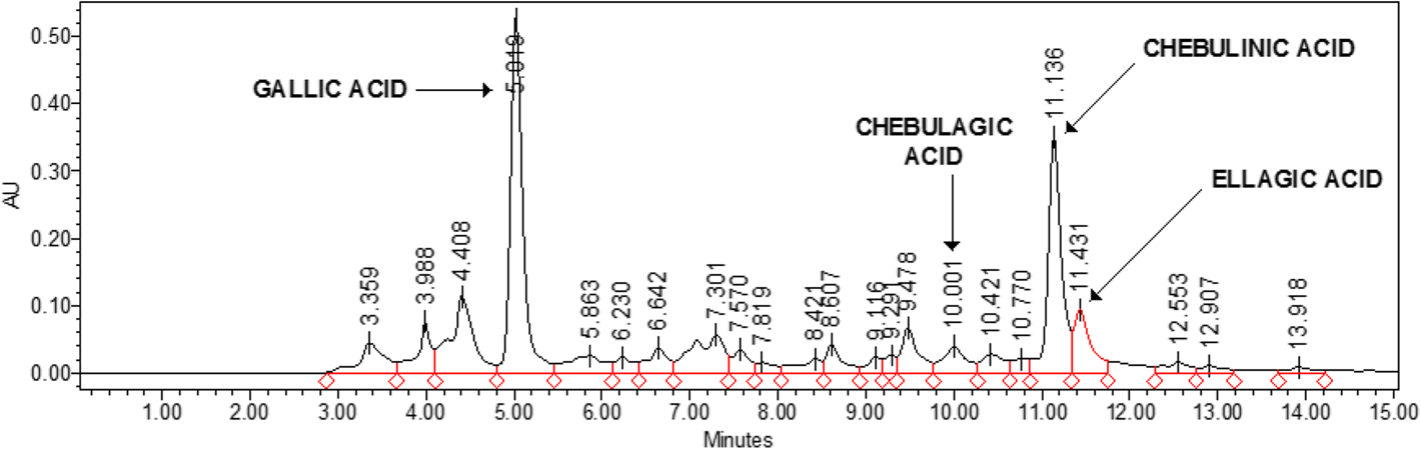
HPLC Chromatogram of *Terminalia chebula* aqueous extract (AF)

Chemical analysis of AyuFlex®, a commercially available standardized aqueous extract of *T. chebula* fruit (Natreon Inc., New Jersey, USA) (AF), indicates a phytochemical profile that includes ≥39% low molecular weight hydrolysable tannins, with ≥15% chebulinic and ≥12% chebulagic acid, and flavonoids at 5.2%, and D-glucose + D-fructose sugars at 6.1% as analyzed by HPLC (see Fig. 1).

This specific *T. chebula* fruit extract has been used in the studies cited below, as well as in the current study. Additional double-blind, placebo-controlled trials have shown that oral administration of *T. chebula* fruit extract (AF) at 250 mg and 500 mg twice daily successfully reduced pain and joint discomfort compared to placebo, with statistically significant improvements in pain threshold force and time, and pain tolerance force and time (*P* < 0.001) [18] and reductions in mWOMAC and knee swelling index, and visual analog scale scores of pain, stiffness, and disability [19]. However, prior to this present study, the AF product had yet to be studied for dose-response effects in healthy subjects without advanced musculoskeletal pathology, and under a more rigorous design with a placebo lead-in.

The primary purpose of this study was to assess the effect of dietary supplementation with *T. chebula* fruit extract (as AyuFlex®) at two different doses compared to placebo over 12-weeks on joint comfort, stiffness, and function utilizing validated questionnaires in healthy subjects with exercise-induced joint discomfort. Secondary and tertiary outcomes included anchored VAS psychometrics to assess effects on self-reported overall joint and spine health, functional capacity with a 6-min walk and leg extension resistance exercise challenge, and biomarkers of inflammation + cartilage/connective tissue metabolism.

## Methods

### Study design

This study was a single-center, randomized, double-blind, placebo-controlled, parallel-groups design conducted at The Center for Applied Health Sciences (Stow, OH). The study was conducted in accordance with the International Conference on Harmonization/Good Clinical Practice Guidelines and the 1996 Declaration of Helsinki. The protocol was reviewed, and all procedures approved by an independent, external, FDA-audited, Institutional Review Board (IntegReview, Austin, TX; October 29th, 2015) and the study was registered at Clinicaltrials.gov NCT02589249 on October 26, 2015, and closed out February 7, 2017. All subjects provided written informed consent to participate prior to commencing any study-related activities.

Once cleared through screening, subjects (*n* = 105 healthy overweight men and women) were enrolled, and randomized into one of three parallel groups to participate in the study that ran over a 14-week period. Subjects were ranked according to bodyweight, placed into block groups of 3 and then randomly assigned in a 1:1:1 ratio to one of three groups (A, B or C). The website: https://www.randomizer.org was used to generate a random allocation sequence into groups. All testing took place at the Center for Applied Health Sciences for the duration of the study with the same staff members responsible for the data collection, enrollment of participants, and assignment into groups.

**Fig. 2:**
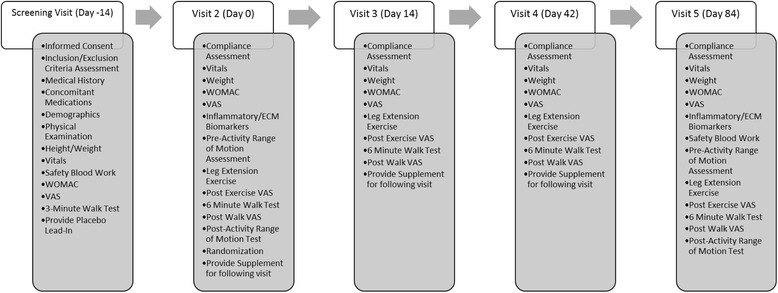
Study Progression

Upon enrollment (Day −14), all subjects completed a two-week placebo lead-in period to enhance statistical power. After successfully completing the lead-in, subjects were randomly assigned (Day 0) to one of three groups (500 mg/day AF, 1000 mg/day AF, Placebo). After randomization, subjects reported to the testing facility at various intervals (Day 14, Day 42, and Day 84) for testing. At each visit, measurements of joint health and function, as well as functional capacity were performed. Measurements of inflammation and extracellular connective tissue matrix were made following the two-week placebo lead-in and following 12-weeks of supplementation (Day 84). Biomarkers of safety were measured at screening and following 12-weeks of supplementation. All blood samples were obtained after a 10 h overnight fast from an antecubital vein by a Research Nurse using standard aseptic phlebotomy procedures (See Fig. 2 for an overview of the study).

### Study subjects

In order to qualify for the study, male or female subjects needed to be ≥35 to ≤70 years of age healthy, overweight adults free of major medical conditions with knee discomfort associated with exercise or physical activity at any time over the 2 weeks prior to screening of at least 30 mm out of 100 mm on a VAS. The National Institutes of Health (NIH) classification system of BMI (body mass index) was utilized to determine whether subjects qualified as overweight (i.e. BMI = 25–29.9). Exclusionary criteria included: diagnosis of an inflammatory joint disorder, osteoarthritis, anemia, previous knee or hip replacement surgery, history or clinically significant gastrointestinal disorders, active neurological, endocrine, cardiovascular, pulmonary, hematological, immunologic/ autoimmune, psychiatric, or metabolic diseases, a history of alcohol or substance abuse, or subjects with a physical disability that would interfere with the functional performance measures.

Potential subjects also were excluded if they were smokers, taking a non-steroidal anti-inflammatory drug, known anti-inflammatory supplements (e.g., Boswellia, curcumin, omega-3 fatty acids, glucosamine, chondroitin, MSM, or collagen), or had a history of allergic reaction or known sensitivity to *T. chebula* or other chemically related botanical/herbal products or supplements. Subjects who received any glucocorticoid (corticosteroid) injection, hyaluronic acid injection, prolotherapy, or PRP (platelet rich plasma) injection, bone marrow or other regenerative injection in either knee within 6 months prior to enrollment were also excluded. Female subjects were excluded if they were pregnant, nursing, or planning on becoming pregnant (See Tables 1 and 2 for details).

**Table 1:** Exclusion criteria

● Subjects with an established diagnosis of inflammatory joint disorder or osteoarthritis per ACR (American College of Rheumatology) guidelines.
● Subjects with a history of knee or hip joint replacement surgery, or any hip or back pain which interferes with walking or exercise testing utilized throughout the study.
● Smoking or tobacco use
● Currently taking, or chronic use within 30 days of anti-inflammatory supplements, Boswellia, Curcumin, Omega-3 fatty acids, Glucosamine, Chondroitin, MSM, or Collagen supplements of any type.
● Daily use of NSAIDs (non-steroidal anti-inflammatory drugs); however, daily use of 81 mg of aspirin (not >81 mg) for cardioprotection is allowed.
● Upon physical screening by the medical staff, any subject with signs of overt nutrient deficiencies or metabolic abnormalities such as anemia. This will also need to be included in the screening assessment.
● Glucocorticoid (Corticosteroid) injection, hyaluronic acid injection, prolotherapy, or PRP (platelet rich plasma) injection, bone marrow or other regenerative injection in affected knee within 6 months prior to enrollment in study.
● Individual has any recent illness or condition (within 6 months of screening) that the Investigator believes would interfere with his or her ability to provide informed consent, comply with the study protocol, or might confound the interpretation of the study results or put the person at undue risk.
● Known or suspected pregnancy, planned pregnancy, or lactation.
● If the subjects has been treated for any psychiatric illness or hospitalized for such within the past year, upon PI discretion, will be excluded from the study.
● History of allergic reaction or known sensitivity to *Terminalia chebula* or other chemically related botanical/ herbal products or supplements.
● Any food allergy, intolerance, restriction or special diet that, in the opinion of the Investigator, could contraindicate the subject’s participation in this study.
● Vital sign abnormalities (seated, resting systolic blood pressure lower than 90 or higher than 150 mmHg, diastolic blood pressure lower than 50 or higher than 100 mmHg, or heart rate less than 50 or more than 110 bpm) at screening.
● History or clinically significant gastrointestinal disorder, (eg, inflammatory bowel diseases), presence of any gastrointestinal pathology, persistent gastrointestinal symptoms (eg, diarrhea, vomiting), liver or kidney disease, gastric bypass, gastric stapling, use of Lapband, or other conditions known to interfere with the absorption, distribution, metabolism, or excretion of dietary supplements.
● History of active neurological, endocrine, cardiovascular, pulmonary, hematological, immunologic/ autoimmune, psychiatric, or metabolic disease that is considered clinically significant by the PI.
● Recent history of (within past 12 months), or strong potential for, alcohol or substance abuse. Alcohol abuse will be defined as >14 drinks per week (1 drink = 12 oz. beer, 5.0 oz. wine, or 1.5 oz. distilled spirits).
● Exposure to any investigational agent or drug product within 30 days prior to study entry.
● Subjects who have any physical disability which could interfere with their ability to perform the functional performance measures included in this protocol.
● Individual has a condition the Investigator believes would interfere with the ability to provide informed consent or comply with study instructions, or that might confound the interpretation of the study results or put the patient at undue risk.

**Table 2:** Inclusion criteria

● Healthy male or female volunteers ≥35 to ≤70 years of age.
● Able to understand study procedures and provide signed informed consent, and authorizes release of relevant health information to study investigator.
● Willing to maintain current background dietary and physical activity pattern throughout study period.
● Normally active and otherwise judged to be in good health on the basis of medical history and physical examination.
● Knee joint:
○ No knee joint discomfort at rest.
○ Experience knee joint discomfort with activity or exercise within the last 2 weeks of at least 30 mm out of 100 mm on VAS rating for “knee discomfort with activity or exercise at any time over the last 2 weeks”.
○ Must achieve a rating of at least 30 mm on a 100 mm VAS at any point throughout the standardized lower extremity exercise performance screening test (Screening test = 3 sets of 10–12 repetitions on seated knee extension machine +3-min walk test at maximal walking velocity).
● Females:
● Non-pregnant, non-lactating females who agree to use effective contraceptive methods throughout the course of the study.
● Females of childbearing potential must agree to use one of the following acceptable birth control methods:
○ Surgically sterile (hysterectomy or bilateral oophorectomy);
○ Surgically sterile (bilateral tubal ligation with surgery at least 6 weeks prior to study initiation)
○ Intrauterine device (IUD) in place for at least 3 months
○ Abstinence (not having sexual intercourse)
○ Barrier method (condom or diaphragm) with spermicide for at least 14 days prior to screening and through study completion

**Fig. 3:**
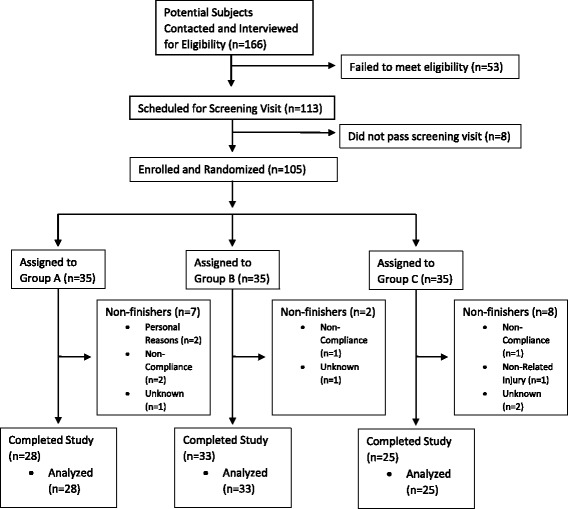
Enrollment and randomization flow chart

A total of 166 potential subjects were recruited and contacted for participation from the population of northeast Ohio using flyers, word-of-mouth, and an existing database of previous studies (which contains ~10,000 subjects). Subjects were initially contacted by telephone and email prior to being interviewed and screened**.** Through telephone screenings, 53 were eliminated due to not meeting eligibility requirements, and eight were ruled ineligible during or following the screening visit. 105 subjects were subsequently enrolled following the results of the blood work from their screening visit (Fig. 3). For this three group, parallel-design prospective study, we used effect sizes and variances from Nutalipati et al. 2016 [19], as well as our own pilot data to calculate our sample size. Thus, we calculated that approximately 90 subjects (30 per group) would be required to detect a between-group difference of 7 units on mWOMAC, and avoid a Type I error using an alpha level of 0.05, and power of 80% as reasonable for avoiding a Type II error.

### Control of diet, exercise training, and physical activity

After they were officially enrolled into the study, a research dietitian met with each subject and explained the proper procedures for recording dietary intake (i.e. determining serving sizes, noting time of day, method of preparation, etc). Each subject’s baseline diet (3 days: two weekdays and one weekend day) was analyzed via NutriBase IV (Clinical Edition, AZ) to determine its energy and macronutrient content. Additional 3-day diet records were analyzed during the last day of testing (Day 84) to verify that eating habits remained consistent throughout the study. In addition, 24-h prior to each visit to the laboratory, subjects duplicated their diets from records obtained at their baseline visit.

Subjects were asked to maintain their usual physical activity patterns during the study. Each subject’s physical activity was assessed via the Yale Physical Activity Questionnaire at baseline (prior to enrollment), and at the last day of testing (Day 84).

### Supplementation

After qualifying for the study, subjects were assigned to receive, in a double-blinded manner, 36 doses of placebo for the first 14-days of the study after screening and enrollment (i.e. placebo lead-in period). Thereafter, subjects were randomized to receive one of three test product treatments in a double-blinded fashion: AF 250 mg BID (500 mg/day), AF 500 mg BID (1000 mg/day), or Placebo BID. Subjects were given enough test product to consume 1 dose twice daily (with breakfast and dinner) until Day 14 and Day 42, respectively. During Visit #4, subjects were given the remainder of their respective test product to finish out the study. HPLC analysis of commercial batch AYF-110315 of the test product AyuFlex®, a standardized aqueous extract of *T. chebula* fruit (commercial product from Natreon Inc., New Jersey, USA) (AF) was confirmed to contain 58% tannins, with 33.8% chebulinic acid, 9.4% chebulagic acid, 9.4% gallic acid, 3.4% elagic acid, 5.2% total flavonoids, 6.1% sugars as D-glucose and D-fructose. Both AF and Placebo capsules included microcrystalline cellulose, croscarmellose sodium (24 mg), silicon dioxide (6 mg) and magnesium stearate (6 mg) as excipients. Placebo capsules contained microcrystalline cellulose at 400 mg, while AF 250 mg capsules contained 300 mg of microcrystalline cellulose and AF 500 mg capsules contained 109 mg microcrystalline cellulose. Both AF test capsule product and placebo capsules were provided by Natreon, Inc.

Compliance to the supplementation protocol was monitored by having subjects complete a daily supplementation (check off) log. In addition, study participants were required to return their supplement containers for pill counts and were reminded of details associated with the study protocols with weekly text messages and/or emails.

### Outcome variables

#### Measures of comfort, mobility and function

Determination of self-reported feelings of joint health, comfort, mobility, and function were the primary outcome measures at every study visit, and were assessed with the modified Knee Injury and Osteoarthritis Outcomes Score (mKOOS) global score, modified Western Ontario and McMaster Universities Arthritis Index (mWOMAC) subscale and several 100 mm Visual Analog Scales (VAS) [20, 21]. The global mKOOS test is composed of four categories (Symptoms, Stiffness, Discomfort, and Function/Daily Living Activities); possible scores range from 0 for individuals with maximum discomfort or functional challenges to 100 for no issues with discomfort or mobility whatsoever (higher scores are favorable). Additionally, mWOMAC subscale scores consist of 24 items divided into three subscales assessing discomfort (5 items), stiffness (2 items) and physical function (17 items).

The anchored 100 mm VAS were utilized to acquire psychometric, self-reported data that represented their feelings over that day and the previous week based on two diametric descriptions [22, 23]. Subjects were asked to rate their feelings of: level of discomfort in the right knee with activity/exercise (No Discomfort At All; Most Discomfort Possible), level of discomfort in the left knee with activity/exercise (No Discomfort At All: Most Discomfort Possible); overall knee mobility (No Motion At All: Greatest Motion Possible); overall arm, hip, and leg function (No Function At All: Greatest Function Possible); low back health (Worst Possible: Best Possible); and willingness to exercise (Lowest Possible: Highest Possible).

#### Joint mobility

After obtaining measurements of Comfort, subjects’ voluntary range of motion was measure at both knee joints. Measures of flexion and extension were measured in both standing and supine (laying) positions. Subjects were instructed to bring each knee comfortably through the range of motion that allowed them to move without experiencing pain or discomfort. An Elite Medical Instruments™ 8-in. goniometer was centered at the subjects’ lateral epicondyle of the femur, and then the angle of the knee joint was measured using the greater trochanter of the femur and the lateral malleolus of the fibula as anatomical alignment points. These measures were taken prior to exercise sessions and again following exercise sessions on Visits 2 and 5.

#### Functional capacity

As a measure of functional capacity, subjects performed six sets of bilateral leg extensions using 30% of each subjects’ respective bodyweight at baseline. Subjects performed 10–12 repetitions per set with 90 s of rest between sets. Subjects were free to conclude the exercise before the final repetition if they experienced joint pain/discomfort that they felt prevented them from continuing the exercise. Total repetitions were recorded. Immediately following the exercise bout, subjects were given a 100 mm anchored VAS scale and asked to rate their “level of right knee discomfort” and “level of left knee discomfort”.

After a three-minute rest period, subjects began a 6-min walk test. The testing took place on a flat-carpeted hallway measuring 48.9 m in length within the medical research facility. Subjects were instructed to cover as much distance as possible in the 6-min time period, while walking. A member of the research staff followed behind measuring the distance using a US-Tape DW-1000™ distance-measuring wheel to determine the distance covered. At the conclusion of the walk, subjects were again given a 100 mm anchored VAS scale and asked to rate “level of right knee discomfort” and “level of left knee discomfort”.

#### Inflammation and safety

Biomarkers of inflammation (high sensitivity C-reactive protein [CRP], tumor necrosis factor-alpha [TNF-alpha]) and extracellular connective tissue matrix turnover (Human Cartilage Oligomeric Matrix Protein [COMP]) were assessed on Visits 2 and Visit 5 from venous blood samples collected from a forearm vein. CRP was measured using Quantitative Latex Immunoturbidimetry by Laboratory Corporation of America®. Immunoturbidimetric assay methods for quantitative determination of hs-CRP have been described [24]. TNF-alpha and COMP were measured via enzyme-linked immunosorbent assay kit from R&D Systems, Bio-Techne Corporation (Minneapolis, MN) per manufacturer’s protocol according to Lai et al. [25]. Coefficients of variation for COMP and TNF-alpha were <5%.

Biomarkers of safety were measured during the screening visit and again following 12-weeks of supplementation. Standard safety profiles (i.e., Comprehensive Metabolic Panel, Complete Blood Count, and Lipid Panel) were assayed by Laboratory Corporation of America®. Treatment-dependent adverse events (AEs) and side-effects were coded by MedDRA® (Medical Dictionary for Regulatory Activities). Subjects were contacted on a weekly basis via email or telephone and queried for AEs.

### Statistical analyses

Data from this study were independently entered into two separate EXCEL spreadsheets. The spreadsheets were compared for discrepancies prior importing the final data into SPSS (IBM® Version 24) for analyses. Statistical analyses were completed by a statistician who was initially blinded to group assignments (i.e. AF 250 mg BID vs. AF 500 mg BID vs. Placebo BID). Following un-blinding, comparisons were made combining active groups (AF 250 mg BID & AF 500 mg BID) vs. Placebo. All endpoints were compared using a two-factor ANCOVA (product dose/placebo [3 levels] x time [4 levels]) using Visit 2 (Day 0) values as the covariate, and delta scores calculated for differences between Day 84 and baseline (Day 0). In all analyses, differences were considered statistically significant when the probability of chance occurrence was less than 5% (i.e. *P* < 0.05), while trends were identified as values of 0.06 ≤ *P* ≤ 0.10. Effect size was determined using partial eta-squared (n_p_^2^) with 0.01 considered to be small, 0.06 to be medium, and 0.14 to be large.

## Results

Table 3 displays the baseline characteristics of the subjects. No statistically significant differences were noted between the groups at baseline in age, height, weight, body mass index, blood pressure, or heart rate. In addition, there were no differences in baseline energy intake (AF1 = 1345 ± 449; AF2 = 1449 ± 547; PBO = 1483 ± 658 kcal; *p* = 0.641), carbohydrate intake (AF1 = 148 ± 65; AF2 = 158 ± 63; PBO = 176 ± 90 g/day; *p* = 0.507), protein intake (AF1 = 71 ± 28; AF2 = 85 ± 47; PBO = 72 ± 29 g/day; *p* = 0.954), fat intake (AF1 = 52 ± 27; AF2 = 53 ± 29; PBO = 55 ± 33 g/day; *p* = 0.225) at baseline or over the course of the study. Similarly, there were no differences in physical activity levels at baseline or over the course of the study (AF1 = 38 ± 7; AF2 = 41 ± 9; PBO = 40 ± 7; *p* = 0.462).

**Table 3:** Subject baseline characteristics

Characteristics	AyuFlex 1 (250 mg BID)	AyuFlex2 (500 mg BID)	Placebo	*p-value*
Sex (*m/f)*	15/13	11/22	11/14	0.288
Age	42.0 (±12.5)	42.8 (±10.1)	45.8 (±11.6)	0.453
Height (cm)	170.2 (±9.3)	172.0 (±10.6)	173.6 (±11.6)	0.495
Weight (kg)	86.5 (±15.9)	89.8 (±15.7)	88.1 (±18.0)	0.734
BMI	29.9 (±5.3)	30.2 (±4.7)	29.1 (±4.2)	0.638
Systolic Pressure (mm Hg)	125.0 (±11.8)	127.8 (±11.5)	127.8 (±10.3)	0.566
Diastolic Pressure (mm Hg)	79.9 (±6.5)	80.5 (±8.1)	79.8 (±8.2)	0.914
Heart Rate (bpm)	70.8 (±8.9)	70.6 (±10.7)	71.7 (±8.6)	0.903

Values are Mean (± Standard Deviation)

### Measures of comfort, mobility and function

#### mKOOS scores

Significant differences (*p* = 0.042; n_p_^2^ = 0.037) were noted when combined Active Groups (AyuFlex 1 + AyuFlex 2) were compared to Placebo over the study duration (Table 4).

**Table 4:** mKOOS scores

Visit	AyuFlex1 (*n = 28)*	AyuFlex2 (*n = 33)*	Placebo (*n* = 25)	Overall^a^	AF1 + AF2 vs PBO	AF1 vs PBO	AF2 vs PBO	AF1 vs AF2
				0.115	***0.042***	0.140	0.620	0.485
0	73.6 (±15.0)	74.0 (±16.0)	74.5 (±14.1)					
14	77.7 (±14.0)	76.5 (±17.4)	74.8 (±14.2)		0.970	0.637	0.635	0.321
42	79.8 (±12.4)	78.8 (±16.8)	75.6 (±15.8)		0.301	0.222	0.526	0.510
84	83.7 (±12.6)	80.9 (±15.3)	77.0 (±16.4)		0.083	***0.036***	0.303	0.227
Changes to mKOOS Scores^b^								
	10.1 (±13.2)	6.9 (±9.9)	2.5 (±8.2)	***0.039***	***0.023***	***0.011***	0.247	0.247

^a^Overall *p* value using mean changes over time amoung the listed group/s using a Repeated Measures ANCOVA

^b^This was determined using Post Score Subtracted from Day O

Values Presented as Mean ± Standard Deviation

Values determined to be significant (*p = <0.05)* ***indicated***

**Fig. 4:**
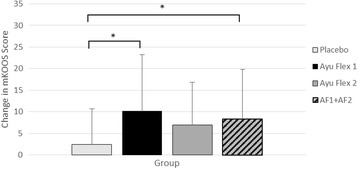
Mean ± SD changes from Day 0 in mKOOS scores over the course of the study (i.e. higher delta scores indicate greater improvement). Compared to the placebo group, both AF1 (*p =* 0.011) and AF1 + AF2 (*p =* 0.023) noted greater improvements

When comparing the change from the baseline visit (Day 0) to the end of the study (Day 84), a significant overall difference (*p =* 0.039) was seen between groups (Table 4). Significant differences (*p = 0.011*) were also seen between AyuFlex 1 and the Placebo group and when both Active Groups were combined vs. Placebo (*p* = 0.023), Fig. 4.

### Changes in mKOOS scores

#### Global mWOMAC scores and subscales

**Fig. 5:**
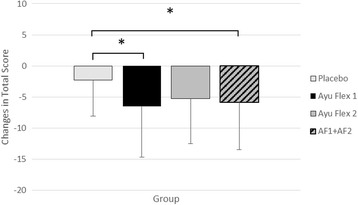
Mean ± SD changes in Physical Function in the modified WOMAC score subscale. Significant differences were noted between the AF1 Group (*p =* 0.039) and the combined Active Groups (*p =* 0.046) vs. the Placebo Group

The modified WOMAC (Physical Function) subscale revealed significant differences between AyuFlex 1 (*p =* 0.039) and the combined Active Groups (*p =* 0.046) vs. the Placebo Group (Fig. 5).

#### Changes to physical function mWOMAC subscale

**Fig. 6:**
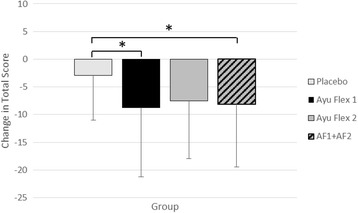
Mean ± SD changes in Total mWOMAC scores. Significant differences were noted between the AF1 group (*p =* 0.047) and when the two Active Groups (*p =* 0.042) were combined vs. the Placebo group

Examining changes in total mWOMAC scores revealed significant differences in the combined Active Groups (*p =* 0.042) and the AyuFlex1 (*p =* 0.047) group when compared to the Placebo Group (Fig. 6).

### Changes to Total mWOMAC score

#### VAS scores

**Fig. 7:**
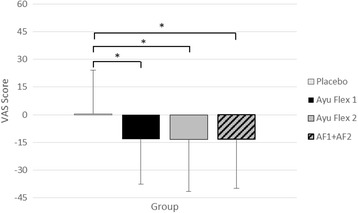
Mean ± SD changes in VAS scores for knee discomfort. Significant differences were noted between the active groups (AF1, AF2, and AF1 + AF2) vs. Placebo. See Table 5 for details

Change scores (Fig. 7) revealed significant differences between the groups (*p =* 0.002) as well as when comparing the Placebo group to both AyuFlex1 (*p =* 0.002), AyuFlex2 (*p =* 0.001), and when the active groups were combined (*p* < 0.001). See Table 5 for details.

**Table 5:** VAS score of measure of discomfort with activity/exercise

Visit	AyuFlex1 (*n = 56)*	AyuFlex2 (*n = 66)*	Placebo (*n = 50)*	*p value*				
				Overall^a^	AF1 + AF2 vs PBO	AF1 vs PBO	AF2 vs PBO	AF1 vs AF2
Overall^a^				0.053	***0.046***	***0.043***	0.105	0.165
0	35.3 (±22.9)	38.2 (±28.0)	31.1 (±22.8)					
14	32.8 (±22.6)	31.3 (±25.8)	33.2 (±21.5)		0.073	0.227	0.036	0.332
42	27.8 (±21.7)	31.4 (±24.5)	32.9 (±24.3)		0.089	0.059	0.263	0.537
84	22.1 (±19.5)	24.7 (±23.8)	33.4 (±25.1)		***0.001***	***0.001***	***0.005***	0.651
Changes to Measures of Discomfort with Activity or Exercise^b^								
	−13.2 (±24.4)	−13.5 (±28.7)	2.2 (±21.6)	***0.002***	***<0.001***	***0.002***	***0.001***	0.956

^a^Overall p value using mean changes over time amoung the listed group/s using a Repeated Measures ANCOVA

^b^This was determined using Post Score Subtracted from Day O

Values Presented as Mean ± Standard Deviation

Values determined to be significant (*p = <0.05)* ***indicated***

### Changes to VAS scores in knee discomfort with activity/exercise

**Fig. 8:**
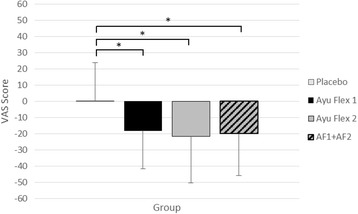
Mean ± SD changes in levels of knee discomfort (subgroup analysis in subjects with scores of 30 mm or higher at the initial screening visit). AyuFlex 1 (*p =* 0.003), AyuFlex 2 (*p = <*0.001), and Combined (AF1 + AF2) (*p* < 0.001) displayed significant differences vs. the Placebo group

A similar subgroup analysis using VAS scores revealed significantly lower scores (Fig. 8) in reported measures of knee pain for the AyuFlex 1 (*p =* 0.003), AyuFlex 2 (*p = <*0.001), and Combined Active (*p* < 0.001) vs. the Placebo Group. There was a significant difference when the Active Groups were combined vs. placebo (*p =* 0.044; np2 = 0.029) during the study (Table 6).

**Table 6:** VAS score of measure of discomfort with activity/exercise adjusted

Visit	AyuFlex1 (*n = 37)*	AyuFlex2 (*n = 39)*	Placebo (*n = 34)*	*p value*				
				Overall^a^	AF1 + AF2 vs PBO	AF1 vs PBO	AF2 vs PBO	AF1 vs AF2
Overall^a^				0.085	***0.044***	0.054	0.082	0.361
0	43.8 (±22.8)	52.0 (±25.7)	40.5 (±20.8)					
14	39.1 (±23.5)	41.7 (±22.8)	40.7 (±20.2)		0.122	0.266	0.090	0.536
42	32.2 (±22.5)	39.4 (±22.9)	39.8 (±22.9)		0.097	***0.050***	0.430	0.414
84	25.7 (±19.0)	30.3 (±21.6)	40.9 (±24.1)		***0.001***	***0.001***	***0.008***	0.595
Changes to Measures of Discomfort with Activity or Exercise^b^								
	−18.2 (±23.5)	−21.7 (±28.6)	0.4 (±24.1)	***0.001***	***<0.001***	***0.002***	***<0.001***	0.547

^a^Overall p value using mean changes over time amoung the listed group/s using a Repeated Measures ANCOVA

^b^This was determined using Post Score Subtracted from Day O

Values Presented as Mean ± Standard Deviation

Values determined to be significant (*p = <0.05)* ***indicated***

### Changes to VAS scores in knee discomfort with activity/exercise adjusted

Secondary measurements of VAS scores for overall knee mobility, overall low back health, and willingness to exercise did not uncover any significant interaction between groups when measured over the course of the study (Table 7). However, overall joint function did reveal significant higher scores from baseline to Day 84 for AyuFlex 2 (*p* = 0.011) and the combined mean change scores of AyuFlex 1 + AyuFlex 2 active groups (*p* = 0.029) vs. Placebo, Table 7.

**Table 7:** VAS secondary measures

Visit	AyuFlex1 (*n = 28)*	AyuFlex2 (*n = 33)*	Placebo (n = 25)	*p value*				
		***Overall Knee Mobility***		Overall^a^	AF1 + AF2 vs PBO	AF1 vs PBO	AF2 vs PBO	AF1 vs AF2
Overall^a^				0.577	0.552	0.344	0.946	0.341
0	69.4 (±21.2)	69.4 (±17.6)	68.8 (±21.8)					
14	68.0 (±22.1)	75.8 (±16.2)	68.0 (±21.8)		0.324	0.892	***0.048***	0.078
42	75.5 (±16.3)	78.2 (±14.9)	70.1 (±21.6)		0.078	0.249	0.078	0.474
84	79.0 (±16.3)	83.4 (±14.9)	73.3 (±18.8)		0.031	0.208	***0.021***	0.251
Changes to Measures of Overall Knee Mobility^b^								
	9.6 (±21.6)	13.9 (±18.9)	4.6 (±17.9)	0.201	0.115	0.351	0.074	0.391
		***Overall Joint Function***		Overall^a^	AF1 + AF2 vs PBO	AF1 vs PBO	AF2 vs PBO	AF1 vs AF2
Overall^a^				0.444	0.199	0.219	0.367	0.776
0	73.3 (±20.5)	66.6 (±23.8)	72.0 (±20.8)					
14	74.8 (±17.9)	76.0 (±18.0)	72.6 (±20.2)		0.304	0.718	0.158	0.448
42	76.2 (±15.1)	79.0 (±19.0)	72.6 (±22.6)		0.388	0.493	0.059	0.226
84	81.1 (±13.0)	82.2 (±17.9)	72.0 (±20.8)		0.387	0.055	0.015	0.522
Changes to Measures of Overall Joint Function^b^								
	7.8 (±21.1)	15.5 (±24.7)	0.0 (±21.1)	***0.038***	***0.029***	0.215	***0.011***	0.182
		***Overall Low Back Health***		Overall^a^	AF1 + AF2 vs PBO	AF1 vs PBO	AF2 vs PBO	AF1 vs AF2
Overall^a^				0.828	0.630	0.907	0.446	0.732
0	64.2 (±22.9)	64.3 (±26.6)	54.4 (±24.6)					
14	71.8 (±18.3)	68.3 (±23.4)	55.8 (±21.2)		***0.032***	***0.017***	0.153	0.405
42	74.5(±22.5)	72.4 (±23.1)	55.8 (±21.2)		***0.007***	***0.014***	***0.024***	0.626
84	76.6 (±20.9)	76.5 (±23.3)	59.8 (±24.4)		***0.013***	***0.029***	***0.037***	0.978
Changes to Measures of Low Back Health^b^								
	12.5 (±27.1)	12.3 (±19.6)	5.5 (±25.0)	0.480	0.224	0.290	0.285	0.975
		***Willingness to Exercise***		Overall^a^	AF1 + AF2 vs PBO	AF1 vs PBO	AF2 vs PBO	AF1 vs AF2
Overall^a^				0.442	0.204	0.171	0.412	0.748
0	74.6 (±21.2)	73.5 (±23.8)	69.2 (±26.8)					
14	74.3 (±17.5)	76.0 (±20.4)	71.0 (±23.6)		0.687	0.941	0.577	0.553
42	79.4 (±16.0)	77.3 (±26.6)	68.4 (±27.5)		0.129	0.112	0.289	0.766
84	82.7 (±16.8)	82.9 (±20.8)	71.1 (±27.9)		***0.039***	0.108	0.082	0.861
Changes to Measures of Willingness to Exercise^b^								
	8.1 (±21.9)	9.5 (±19.6)	2.0 (±27.0)	0.434	0.203	0.331	0.215	0.809

^a^Overall p value using mean changes over time amoung the listed group/s using a Repeated Measures ANCOVA

^b^This was determined using Post Score Subtracted from Day O

Values Presented as Mean ± Standard Deviation

Values determined to be significant (*p = <0.05)* ***indicated***

### Joint mobility

No differences were observed between goniometric range of motion measurements between the groups or when combining the treatment groups vs. the Placebo (data not shown).

### Functional capacity

#### Leg extension and VAS response

**Fig. 9:**
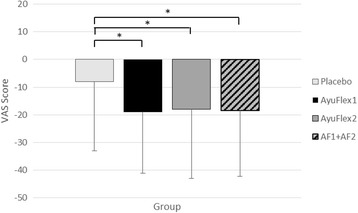
Mean ± SD changes in knee pain following leg extension exercise. Significant differences were noted between the AyuFlex 1 (*p =* 0.022), AyuFlex 2 (*p =* 0.043), and AF1 + AF2 (*p =* 0.039) vs. Placebo

In terms of performance, the number of leg extension repetitions subjects were able to complete did not differ between groups over time. However, differences were observed in both the AyuFlex 1 (*p =* 0.022), AyuFlex 2 (*p =* 0.043), and Combined Active Groups (*p =* 0.039) (Table 8) vs. Placebo for changes in the level of knee pain following the leg extension exercise bout (Fig. 9).

**Table 8:** VAS scores following leg extension

Visit	AyuFlex1 (*n = 56)*	AyuFlex2 (*n = 66)*	Placebo (n = 50)	*p value*				
				Overall^a^	AF1 + AF2 vs PBO	AF1 vs PBO	AF2 vs PBO	AF1 vs AF2
Overall^a^				0.125	***0.046***	0.258	***0.031***	0.584
0	4.1 (±2.5)	4.2 (±3.3)	3.9 (±2.6)					
14	3.6 (±2.3)	4.2 (±3.3)	3.7 (±2.8)		0.966	0.409	0.463	0.088
42	3.0 (±2.3)	3.4 (±3.0)	3.6 (±2.6)		0.122	***0.045***	0.392	0.292
84	2.2 (±2.1)	2.3 (±2.9)	3.1 (±2.5)		***0.010***	***0.010***	***0.033***	0.668
Changes to Measures of VAS Score Following Leg Extension^b^								
	−1.9 (±2.2)	−1.8 (±2.5)	−0.8 (±2.5)	***0.048***	***0.039***	***0.022***	***0.043***	0.717

^a^Overall p value using mean changes over time amoung the listed group/s using a Repeated Measures ANCOVA

^b^This was determined using Post Score Subtracted from Day 0

Values Presented as Mean ± Standard Deviation

Values determined to be significant (*p = <0.05)* ***indicated***

### Changes to knee pain following leg extension exercise

#### 6-min walk test and VAS response

**Fig. 10:**
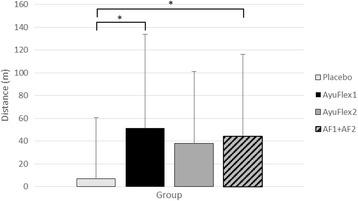
Mean ± SD changes in the distance covered during the 6-min walk test. Significant differences were noted between the AyuFlex 1 group (*p =* 0.019) and the combined Active Groups (AF1 + AF2) (*p =* 0.022) vs. Placebo

Significant improvements in the distance covered during the 6-Minute Walk Test were noted between AyuFlex 1 group (*p* = 0.019) and when combining the two Active Groups vs. the Placebo Group (*p* = 0.022) (Fig. 10).

### Changes to distance covered during 6-minute walk test

**Fig. 11:**
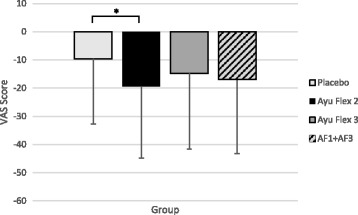
Mean ± SD changes in reported levels of discomfort/pain following the 6-Minute Walk Test. A significant (*p =* 0.048) difference was noted between AyuFlex 1 and the Placebo

Knee pain/discomfort following the 6-Minute Walk Test tended to be different between groups over time (*p =* 0.087). Post hoc testing revealed a significant difference (*p* = 0.048) between AF1 vs. Placebo (Fig. 11).

### Changes to VAS score post 6-minute walk test

#### Inflammation and safety biomarkers

**Fig. 12:**
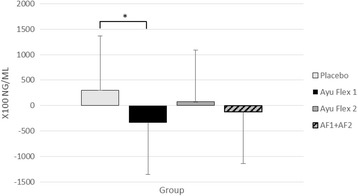
Mean ± SD changes in serum concentrations of COMP. A significant difference was noted between AF1 vs. Placebo (*p =* 0.033)

Measures of COMP tended (*p =* 0.091; n_p_^2^ = 0.061) to interact over time between groups. Post hoc testing revealed a significant difference between AyuFlex 1 and Placebo (*p =* 0.033) (Fig. 12). No significant differences (*p = 0.104*) were noted between the combined Active Groups (AF1 + AF2) and the Placebo Group (Table 9).

**Table 9:** Measures of inflammatory markers

Variable	Day 0	Day 84	Within Group *p*-Value	Between Group *p*-Value
Human COMP (×100 ng/mL)				
Placebo	4280.4 (±1037.8)	4577.8 (±1498.7)	0.195	0.091
AF1	4151.0 (±1186.6)	3818.9 (±1171.4)	0.092	
AF2	4229.5 (±1339.9)	4303.0 (±1387.6)	0.700	
AF1 + AF2	4191.7 (±1258.4)	4069.6 (±1296.4)	0.371	
TNF-alpha (pg/mL)				
Placebo	38.9 (±26.3)	37.2 (±22.2)	0.733	0.492
AF1	51.7 (±35.6)	49.5 (±37.3)	0.641	
AF2	47.5 (±26.3)	39.4 (±25.5)	**0.026**	
AF1 + AF2	49.5 (±30.1)	44.3 (±31.9)	0.074	
C-Reactive Protein (mg/L)				
Placebo	2.5 (±1.8)	1.7 (±1.5)	0.044	0.137
AF1	2.5 (±2.7)	2.5 (±3.1)	0.909	
AF2	2.3 (±2.2)	2.0 (±1.9)	0.249	
AF1 + AF2	2.3 (±2.4)	2.2 (±2.5)	0.619	

Values Presented as Mean ± Standard Deviation

Values determined to be significant (*p = <0.05)* ***indicated***

### Changes to COMP

Although several within-group changes occurred over time in metabolic measures (hematocrit, albumin, ALT), plasma chloride was the only clinical chemistry marker that changed significantly (*p = 0.033*) between groups during the study (Table 10).

**Table 10:** Changes in metabolic measures from day −14 to day 84

Variable	Day −14	Day 84	Within Group *p*-Value	Between Group p-Value
Hematocrit (%)				
Placebo	14.7 (±1.4)	14.7 (±1.4)	0.539	0.787
AF1	14.4 (±1.2)	14.2 (±1.1)	0.058	
AF2	14.7 (±1.2)	14.5 (±1.1)	0.052	
AF1 + AF2	14.6 (±1.2)	14.3 (±1.1)	0.006	
White Blood Cell (x10E3/uL)				
Placebo	6.4 (±1.2)	6.3 (±1.8)	0.747	0.929
AF1	5.7 (±2.0)	5.8 (±1.9)	0.826	
AF2	5.7 (±1.8)	5.7 (±2.0)	0.984	
AF1 + AF2	5.7 (±1.9)	5.7 (±1.9)	0.900	
Potassium (mmol/L)				
Placebo	4.3 (±0.2)	4.3 (±0.3)	0.608	0.870
AF1	4.3 (±0.2)	4.4 (±0.2)	0.581	
AF2	4.3 (±0.3)	4.4 (±0.2)	0.203	
AF1 + AF2	4.3 (±0.2)	4.4 (±0.2)	0.182	
Albumin (g/dL)				
Placebo	4.4 (±0.3)	4.5 (±0.3)	0.682	0.222
AF1	4.4 (±0.3)	4.4 (±0.3)	0.311	
AF2	4.4 (±0.3)	4.4 (±0.2)	0.049	
AF1 + AF2	4.5 (±0.3)	4.4 (±0.3)	***0.003***	
Sodium (mmol/L)				
Placebo	140.5 (±2.1)	140.2 (±1.7)	0.510	0.949
AF1	140.3 (±1.6)	140.1 (±1.7)	0.621	
AF2	141.2 (±2.1)	141.2 (±2.2)	0.829	
AF1 + AF2	140.8 (±1.9)	140.7 (±2.0)	0.622	
BUN (mg/dL)				
Placebo	14.6 (±3.9)	14.3 (±2.8)	0.617	0.402
AF1	13.6 (±3.5)	13.3 (±3.5)	0.604	
AF2	15.3 (±4.1)	14.6 (±4.4)	0.312	
AF1 + AF2	14.5 (±3.9)	14.0 (±4.0)	0.253	
Chloride (mmol/L)				
Placebo	101.7 (±3.9)	102.0 (±2.1)	0.532	***0.032***
AF1	102.9 (±3.0)	101.4 (±2.1)	***0.004***	
AF2	102.8 (±2.1)	102.5 (±2.4)	0.484	
AF1 + AF2	102.8 (±2.5)	102.0 (±2.3)	***0.013***	
Creatinine (mg/dL)				
Placebo	0.88 (±0.1)	0.87 (±0.1)	0.513	0.436
AF1	0.84 (±0.2)	0.86 (±0.2)	0.163	
AF2	0.92 (±0.2)	0.92 (±0.2)	0.977	
AF1 + AF2	0.88 (±0.17)	0.89 (±0.18)	0.413	
AST (IU/L)				
Placebo	21.5 (±5.9)	19.9 (±4.0)	0.059	0.615
AF1	21.4 (±9.8)	20.7 (±9.0)	0.499	
AF2	23.0 (±14.8)	22.9 (±11.5)	0.933	
AF1 + AF2	22.3 (±12.7)	21.9 (±10.4)	0.674	
ALT (IU/L)				
Placebo	25.8 (±11.3)	24.4 (±9.6)	0.427	0.617
AF1	23.2 (±11.6)	19.5 (±8.0)	***0.020***	
AF2	27.2 (±15.8)	24.4 (±15.2)	0.082	
AF1 + AF2	25.4 (±14.1)	22.1 (±12.6)	0.620	

Values Presented as Mean ± Standard Deviation

Values determined to be significant (*p = <0.05)* ***indicated***

Total cholesterol significantly decreased in the combined AF1 + AF2 group over time (Table 11).

**Table 11:** Changes in lipid measures from day −14 to day 84

Variable	Day −14	Day 84	Within Group p-Value	Between Group p-Value
Cholesterol (mg/dL)				
Placebo	202.2 (±54.0)	192.1 (±47.8)	0.066	0.787
AF1	198.1 (±59.5)	187.2 (±35.8)	0.153	
AF2	197.0 (±43.3)	190.9 (±39.3)	0.086	
AF1 + AF2	197.5 (±50.9)	189.2 (±37.4)	***0.036***	
Triglycerides (mg/dL)				
Placebo	156.0 (±126.7)	143.4 (±101.7)	0.473	0.465
AF1	112.7 (±60.9)	118.2 (±88.1)	0.626	
AF2	144.4 (±112.1)	129.7 (±80.0)	0.153	
AF1 + AF2	129.9 (±92.9)	124.4 (±83.3)	0.470	
HDL (mg/dL)				
Placebo	55.1 (±17.4)	53.3 (±15.9)	0.209	0.294
AF1	56.5 (±18.9)	55.5 (±19.3)	0.258	
AF2	52.2 (±16.1)	52.8 (±15.7)	0.577	
AF1 + AF2	54.2 (±17.4)	54.0 (±17.3)	0.853	
LDL (mg/dL)				
Placebo	115.1 (±37.4)	110.1 (±38.1)	0.217	0.550
AF1	119.1 (±54.9)	108.0 (±33.4)	0.154	
AF2	119.7 (±32.8)	116.1 (±29.0)	0.254	
AF1 + AF2	119.4 (±43.9)	112.4 (±31.1)	0.072	

Values Presented as Mean ± Standard Deviation

Values determined to be significant (*p = <0.05)* ***indicated***

**Table 12:** Summary of adverse events

	*Ayu Flex 1*	*Ayu Flex 2*	*Placebo*
Severity			
Mild	2	2	1
Moderate		1	
Severe			
Relationship to Study			
Unlikely		1	
Possible	2	2	1
Probable			
Seriousness			
No			
Hospitalization			
Disability			
Life Threatening			
Important Medical Event			
Reported Symptom			
Crepitus	1		
Cramps	1		
Swelling (From Fall)		1	
Headaches		1	
Heart Burn		1	
Reflux			1

## Discussion

The purpose of this study was to determine the effects of a branded form of *Terminalia chebula* fruit extract (AyuFlex®) on joint mobility, joint comfort, and functional capacity in healthy overweight subjects. The results reveal several important findings, including significant improvements in mWOMAC scores, distance covered in the 6-min walk test, and various VAS subscales. In many of the analyses, no difference was noted between the lower dose (250 mg 2× daily) vs. the higher dose (500 mg 2× daily), indicating a lack of dose dependency for these outcome variables. Our results are congruent with previous data on AF in patients with moderate osteoarthritis and pain at rest by Nutalapati et al. [23] for measures of joint discomfort, function and mobility, yet with some important distinguishing contributions to the literature. Our interrogation on AF has added to the existing body of evidence by utilizing healthy subjects without existing joint pain at rest or diagnosed osteoarthritis, exploring dose-response, effects on biochemical markers and other musculoskeletal regions beyond the knee.

The global mKOOS demonstrated a significant improvement when mean data from both dose groups AF1 (250 mg twice daily) and AF2 (500 mg twice daily) were combined relative to placebo over the 12-week treatment period. Although the slope of change is evident for the AF1 + AF2 throughout the study visits preceding Day 84, the analysis of change scores in global mKOOS (aggregate of changes in symptoms, stiffness, discomfort and function with activities of daily living) from baseline demonstrated that AF1 alone (−14%) and AF1 + AF2 (−12%) were both effective at improving the overall score at Day 84 relative to placebo (−3%). The change from baseline to Day 84 in total mWOMAC, and the mWOMAC physical function subscale were both significantly improved in the AF1 group and the combined mean data from AF1 + AF2 groups relative to placebo. The magnitude of change in our study is comparable to what has been reported in other studies using chondroprotective or anti-inflammatory dietary supplements [26–31]. Multiple factors are likely contributing to the discrepancies in primary endpoints of pain with many of these comparative studies such as: the study population (established OA with active pathology and pain at rest), greater starting level of pain and dysfunction due to advanced disease, lack of additional rigorous placebo lead-in period and inclusion/exclusion criteria as part of the study design.

Our data indicates that there is no clear dose-response relationship with the AF doses examined in our study, which is in stark contrast to previous data from Nutalipati et al., where the 500 mg 2× daily group substantially outperformed (89% greater improvement in WOMAC) the 250 mg 2× daily treatment [23] in change in WOMAC scores from baseline. The major divergence in sample population may be the key determinant for these unexpected results, with subjects in the previous AF study presenting with pain at rest and moderate OA during screening/ enrollment. On the other hand, the current study included a cohort of relatively healthy subjects per the inclusion criteria for enrollment, who were free from any pain at rest, and did not have advanced knee pathology associated with OA (per the American College of Rheumatology guidelines). It is clear that linear dose-response effects are expected based on data in moderate to severe, chronic osteoarthritis patients undergoing various therapeutic interventions, from treatment with NSAIDs (non-steroidal anti-inflammatory drugs) to weight loss and exercise in patients [32, 33]. As such, our lack of a linear dose-response effect may be the result of differences in magnitude and progression of the underlying physiology in healthy subjects with no pain at rest, and discomfort only provoked by an exercise challenge, as opposed to the pathophysiology of patients with chronic, degenerative osteoarthritis.

The secondary outcome measures included anchored VAS for qualitative, self-reported assessment of various joint and orthopedic health parameters, such as “knee discomfort with activity/exercise,” and overall joint function revealed multiple additional significant improvements as changes from baseline to Day 84 for AF2. Additionally, upon post-hoc testing, the anchored VAS for knee discomfort with activity/exercise appeared more acutely for combined AF1 + AF2 active groups at Day 14 and Day 42 with effects that were not detected with the primary endpoints of mKOOS and mWOMAC until day 84. The VAS has demonstrated higher sensitivity relative to WOMAC to changes in pain and comfort assessed hourly for up to 4 h, and weekly for up to 4 weeks following therapeutic intervention [34]. This difference in the ability to detect minimal clinically relevant change acutely supports our observations in VAS that were not otherwise apparent sooner on mWOMAC. The work of both Kumar et al. and Pokuri et al. demonstrated potent, acute analgesic threshold and tolerance effects of aqueous Terminalia chebula extract (AyuFlex) at 1000 mg/day in healthy human subjects [22, 35]. Improvements in VAS measures across multiple time points, global mKOOS, and mWOMAC physical function may stem from increased pain threshold and tolerance via central and peripheral mechanisms that include anti-inflammatory COX, LOX, and TNF-a inhibition [36]. Even amongst the other VAS measures [“knee joint mobility”(*p* = 0.074), “overall low back health/wellness” (0.224), “motivation to exercise” (*p* = 0.21)], most of the AyuFlex treatment groups tended to show greater mean improvements from baseline to Day 84, as well as changes over time using the RM-ANCOVA, but failed to reach significance. Research indicates that over 80% of the population will experience LBP at some time during their lifetime [37], and 69% of those suffering from LBP felt that it affected their daily lives [38]. Conventional medical treatment for LBP typically includes the same types of medications as used for OA. Given the overlapping pathophysiology, some of the same potential mechanisms at play with improvements in knee comfort and function may also contribute to more general musculoskeletal effects. These tendencies warrant additional study beyond knee health/function; future investigations should be powered and designed to assess the effects of AF on exercise/activity-induced spine and peripheral joint discomfort.

In the current study, we observed all active groups (AF1, AF2 and combined AF1 + AF2) significantly decreased VAS discomfort post-leg extension resistance exercise at Day 84 from baseline relative to Placebo. Moreover, another critical indicator of functional exercise capacity and impactful activities of daily living, the 6-min walk test showed significant improvements in total distance ambulated and VAS pain post-6-min walk with AF1 at Day 84 from baseline. To put these findings in context, a large number of previous studies assessing the efficacy of single ingredient and multi-ingredient joint health products on objective functional capacity, such as 6-min walk test, failed to show benefit over placebo [25, 39–42].

We observed a significant reduction of cartilage oligomeric matrix protein (COMP), a biochemical marker of connective tissue integrity and cartilage matrix turnover, in the AF1 group relative to placebo. This finding is consistent with a decrease in the net degradation and turnover rate of joint cartilage matrix molecules as a result of treatment with AF. The increase in mean serum concentrations of COMP in the placebo group is indicative of proteolytic cleavage and repair attempts by chondrocytes to keep up with matrix degradation [43].

In this study treatment with AF tended to demonstrate a decrease in CRP relative to placebo from baseline to day 84, but failed to reach significance. Additionally, AF2 demonstrated a within-group decrease in serum TNF-alpha levels from baseline to day 84. AF contains hydrolysable tannins such as chebulagic acid, chebulinic acid and gallic acid that have been shown to possess anti-infammatory and anti-oxidative activities. Preclinical arthritic pain models and in vitro studies demonstrate suppression of TNF-alpha, IL-6, NF-kappaB, nitrous free radical scavenging and T-cell mediated cytotoxicity [44–46]. Rani et al. and Kishore et al. have previously noted the efficacy of AF extract at 250 mg and 500 mg twice daily for modulating inflammation and oxidative stress, demonstrating dose-dependent significant decreases in plasma levels of hsCRP and MDA over placebo in type-2 diabetic patients. [Unpublished observations by Rani, PU, Sravanti IV, Fatima N, Muralidhar N, Salomi R., 13].

Significant treatment effects of AF extract observed in our study may be driven by mechanisms that range from mitigating chondro-catabolic, pro-inflammatory cytokines and extracellular matrix (ECM) hydrolytic enzymes, to augmenting chondro-anabolic chemokines that stimulate chrondrocyte and synoviocyte metabolism [29, 47, 48].

Analysis of comprehensive blood chemistries revealed a statistically significant decrease in plasma chloride in the AF1 group relative to placebo and AF2. As the plasma chloride value, along with all other biochemical and hematologic measures remained well within clinical normative ranges, this change is deemed to not be indicative of a clinically meaningful change. Interestingly, AF1 + AF2 groups demonstrated a reduction in total cholesterol levels throughout the supplementation period, and is consistent with reports of reductions in blood lipids in diabetic rat models treated with aqueous extracts of *Terminalia chebula* [49]. We observed no significant change in any other clinical blood markers or resting hemodynamics (blood pressure, heart rate, rate-pressure product). Moreover, reported side effects were not disproportional in any of the treatment AF or placebo groups, nor were any serious adverse events reported (Table 12). Results of the present investigation indicate that AF is well-tolerated and does not adversely affect general markers of health.

Future studies may explore even lower doses since a true dose-response in healthy subjects may be lower than 250 mg twice daily, as well as investigating other applications such as higher intensity exercise-induced muscle and connective tissue damage; and analysis of synovial fluid for molecular markers, proteomics, and transcipritomic analyses. Exclusion criteria and compliance with the study protocol did not allow for the use of joint health dietary supplements, and macronutrient data was collected at intervals throughout the study to assess for any potential differences between groups. However, the use of a multivitamin/multimineral supplement was not assessed prior to enrollment during screening visit, nor throughout the duration of the study. This limitation may add a potential, though low leverage confounder to connective tissue metabolism if any of the subjects had supplemented with a multivitamin/mineral product prior to enrollment or throughout the 12 weeks study. Finally, use of AF in conjunction with other nutraceutical ingredients, as part of multi-ingredient dietary supplement preparations with complementary mechanisms of action for joint and musculoskeletal health and function should be investigated.

## Conclusion

To the best of our knowledge this is the first investigation of an aqueous extract of *Terminalia chebula* utilizing several unique study design aspects, including a placebo lead-in, rigorous inclusion/exclusion criteria, expanding beyond knee joint to other MSK areas as secondary endpoints, and the use of healthy subjects without joint pain at rest. This is in contrast the majority of available human data on nutraceutical agents for joint health/function that are performed in populations with pain at rest with chronic, advanced osteoarthritic pathology and degenerative joint disease. In summary, 84 days of AyuFlex® supplementation improves knee and overall joint health and functional capacity outcomes in otherwise healthy subjects with exercise/activity-dependent knee discomfort. These data also suggest that benefits may extend beyond knee joints to include overall/whole-body joint and spine health.
